# Supplementary material for: Do Not Miss Acute Diffuse Panbronchiolitis for Tree-in-Bud: Case Series of a Rare Lung Disease
Source: Diagnostics (Basel). 2022 Jul 7;12(7):1653. doi: 10.3390/diagnostics12071653 (PMC9323848; doi:10.3390/diagnostics12071653)
Supplement: Supplementary file 1 [file diagnostics-12-01653-s001.zip › diagnostics-1770545-supplementary.pdf]

Supplementary Materials

Patient perspective

A good doctor–patient relationship is essential for honest answers to unpleasant questions, such as possible drug use. In the best case, the patient can be convinced not to use drugs in the future:

"I did not know how sick I was. I realized how important it was for the doctors and nurses to know what I had smoked so that they could work out what was making me so short of breath. It was good to be able to tell them without being judged. I could tell that they really wanted to help me. They told me afterwards that they wished they had thought of more questions before they had to intubate me because my parents couldn't answer all their questions. When I woke up and learned what had made me so sick, there was one thing I was certain of: I definitely didn't want this to happen to me again. On the one hand, I knew that I never wanted to smoke again. On the other hand, I worried about what would happen when I am out with my friends. It was important to talk with my friends and to know they want to support me in not smoking. I had good talks with the doctor and psychologist in the hospital before I went home, thinking through different situations in which I might end up smoking and how to avoid them. Getting into my sport again will help."
